# Association of depressive symptoms and quality of life in Pakistani youth (15–24 years) with polycystic ovarian syndrome: a web-based analytical cross-sectional study

**DOI:** 10.3389/fgwh.2023.967883

**Published:** 2023-06-21

**Authors:** Ghazal Peerwani, Shafquat Rozi, Maryam Pyar Ali Lakhdir, Nadeem Zuberi, Nargis Asad

**Affiliations:** ^1^ Department of Community Health Sciences, Aga Khan University, Karachi, Pakistan; ^2^ Department of Clinical Research Cardiology, Tabba Heart Institute, Karachi, Pakistan; ^3^ Department of Obstetrics and Gynaecology, Aga Khan University, Karachi, Pakistan; ^4^ Department of Psychiatry, Aga Khan University, Karachi, Pakistan

**Keywords:** depressive symptoms, health related quality of life, Pakistan, polycystic ovarian syndrome (PCOS), youth

## Abstract

**Introduction:**

Polycystic ovarian syndrome (PCOS) is associated with impaired quality of life (QOL) of individuals, predominantly in youth, who are most vulnerable to its impact. Psychological morbidity could be one of the factors influencing QOL. The study investigated the association between depressive symptoms and QOL in Pakistani youth (15–24 years) with PCOS and determined other factors associated with QOL.

**Methods:**

We conducted an analytical-cross-sectional survey on 213 single Pakistani females aged 15–24 years recruited via a web-based approach. Depression and QOL were assessed through Center-of-Epidemiological-Studies-Depression tool and Polycystic-ovarian-syndrome-quality-of-life-scale. Multiple-linear-regression was used to determine factors associated with QOL, and adjusted regression-coefficients along with a 95% confidence interval were reported.

**Results:**

The mean QOL score: 2.9 ± 1.1. The domain of obesity had the lowest mean score (2.5 ± 1.6) whereas domain of hirsutism had the highest (3.2 ± 1.9). 172/213 (80%) participants were screened positive for depressive symptoms. Participants with depressive symptoms reported reduced mean QOL scores than respondents with no such symptoms (2.8 ± 1.0 vs. 3.4 ± 1.3, *p* < 0.001). No differences were found in overall QOL and individual domains between participants 15–19 years (*n* = 36, 17%) and participants >19–24 years (*n* = 177, 83%) (2.9 ± 1.1 vs. 2.9 ± 1.1) (*p* > 0.05). We found a significant interaction between depressive symptoms and PCOS duration, indicating that the estimated mean overall QOL score decreases by 25.1 (−36.6, −13.6) for every year increase in PCOS duration among participants screened positive for depressive symptoms. Furthermore, for those respondents who had family history of PCOS and were not satisfied with their healthcare provider treating PCOS, the estimated mean QOL score was 17.47 (−26.1, −8.8) lower than participants who had no family history of PCOS and were satisfied with their healthcare provider. Other factors associated with reduced quality of life included societal pressure to improve appearance affected by PCOS, parental criticism related to PCOS, education, socioeconomic status, working status and BMI.

**Conclusion:**

Depressive symptoms with increasing duration of PCOS were significantly associated with reduced QOL. Therefore, to improve the overall QOL of PCOS youth, screening and timely addressing of psychological morbidities should be considered.

## 1. Introduction

Polycystic ovarian syndrome (PCOS) is one of the most common endocrine disorders encountered by 11% of youth globally (1). This syndrome usually presents itself during adolescence, soon after menarche, and is manifested by hyperandrogenism and ovulatory dysfunction leading to manifestations like amenorrhea, oligomenorrhea, along with physical changes including obesity, alopecia, hirsutism, and acne (2). Clinical symptoms inherent in PCOS cause many distress and concern amongst women, mainly due to their body image and femininity, and are frequently cited as a reason for the disturbed or reduced disease-specific quality of life (QOL) (3). Disease-specific QOL is a dynamic, multifaceted concept which measures the insight of one's physical, social, and emotional aspects and the extent to which it is affected by a specific medical condition, its treatment, and ultimate outcome (4). One of the most common tools used assess disease specific QOL is PCOS-Q (Polycystic Ovarian Syndrome Quality of life). It consists of five domains including emotional health (8 items), menstrual abnormalities (4 items), hirsutism (5 items), infertility (4 items), and obesity (5 items). Various studies have indicated PCOSQ as a reliable measure for assessing QOL in youth (Cronbach alpha 0.7–0.97 reported) (5). Internationally, Obesity/weight and menstrual abnormalities were the most distressing factors impacting QOL in American and Brazilian youth (6, 7). In Bulgaria, hirsutism was the most distressing domain of QOL in youth under 25 (8). Regionally in Iran, infertility and menstrual abnormalities were most significantly affecting the QOL, whereas, in India, emotional health and menstrual abnormalities were the most distressing domains of QOL in unmarried youth with PCOS (9, 10).

There are multiple factors associated with impaired QOL in women with PCOS. Literature cites depression as one of the most significant factors influencing disease-specific QOL in PCOS women. Various studies have reported that depressed women with PCOS had lower QOL scores than those with no depressive symptoms (11, 12). Depression in women with PCOS might change their perspective towards how they perceive their symptoms, ultimately affecting their disease-specific QOL (12); however, this relationship has not been well explored in youth. One of the most used tools to assess depressive symptoms is CES-D (Centre of Epidemiological Studies-Depression)-20. This scale differs from other scales of evaluating depression in a way that it measures the current depressive symptoms status, which is required for screening youth and not just an occurrence of a major depressive episode (13–15). This scale is constructed to be easily used in surveys of all age groups; hence it is designed as a short, structured, self-reported measure. Other potential factors influencing QOL of women diagnosed with PCOS include age, socioeconomic status, education status, societal pressure to improve appearance, confidence in healthcare provider (HCP) to treat PCOS symptoms, parental criticism due to PCOS, family history of PCOS, indulgence in physical activity and BMI (12, 16, 17).

Despite various international and regional studies catering to the consequences of PCOS, the relationship between psychological morbidity and QOL of PCOS women, predominantly single (unmarried) and youth is poorly understood. As per WHO, late adolescence is defined as age 15–19, whereas youth is defined till 24 years of age (18). This is the most vulnerable and understudied age bracket affected by PCOS and its implications because these phases are characterized by extreme consciousness and distinctiveness about the physical appearance. Any deviation from conventional aesthetics might affect the psychological well-being as well as QOL (19). Moreover, the ultimate outcomes of psychological morbidity and poor QOL in young girls, as observed by literature, include suicidal tendencies, co-morbidities, and substance abuse (20) In Pakistan, one-third of the overall 212 million population lies in this age bracket, of which 48% are girls (21, 22). In Pakistan where mental health stigma, especially in this age bracket, is prevalent and the concept of QOL is subdued (23), it becomes necessary to determine the QOL of youth with PCOS and also to identify factors influencing QOL as it is a critical step towards identifying the adequate measures and interventions required to ensure their overall psychological, physical, and social well-being. Furthermore, if the association between depression and QOL is established, then by adequate psychological therapies, the overall QOL of PCOS women can be improved. Thus, we employed a web based approach to determine the association between depressive symptoms and QOL as almost 54% of Pakistani youth has access to internet access. This study investigates the association between depressive symptoms and QOL and also identifies factors affecting QOL of Pakistani youth (15–24 years) with PCOS.

## 2. Methods

A cross-sectional survey using a web-based approach was employed to determine the association between depressive symptoms and QOL in Pakistani youth with internet access between June-August 2021.

The consent and questionnaire of this study were formulated on Google Forms, and the relevant link of the Google Form was disseminated via multiple social media platforms, including Facebook, Instagram, and WhatsApp. Many Facebook and Instagram groups of Pakistani girls and women with PCOS were targeted to recruit participants. Moreover, the link was also sent via WhatsApp to acquaintances, and they were requested to send it to their contacts for snowballing effect. As this study was conducted during the pandemic amidst stringent lockdowns, a web-based recruitment and data collection approach seemed most feasible.

**Figure 1:**
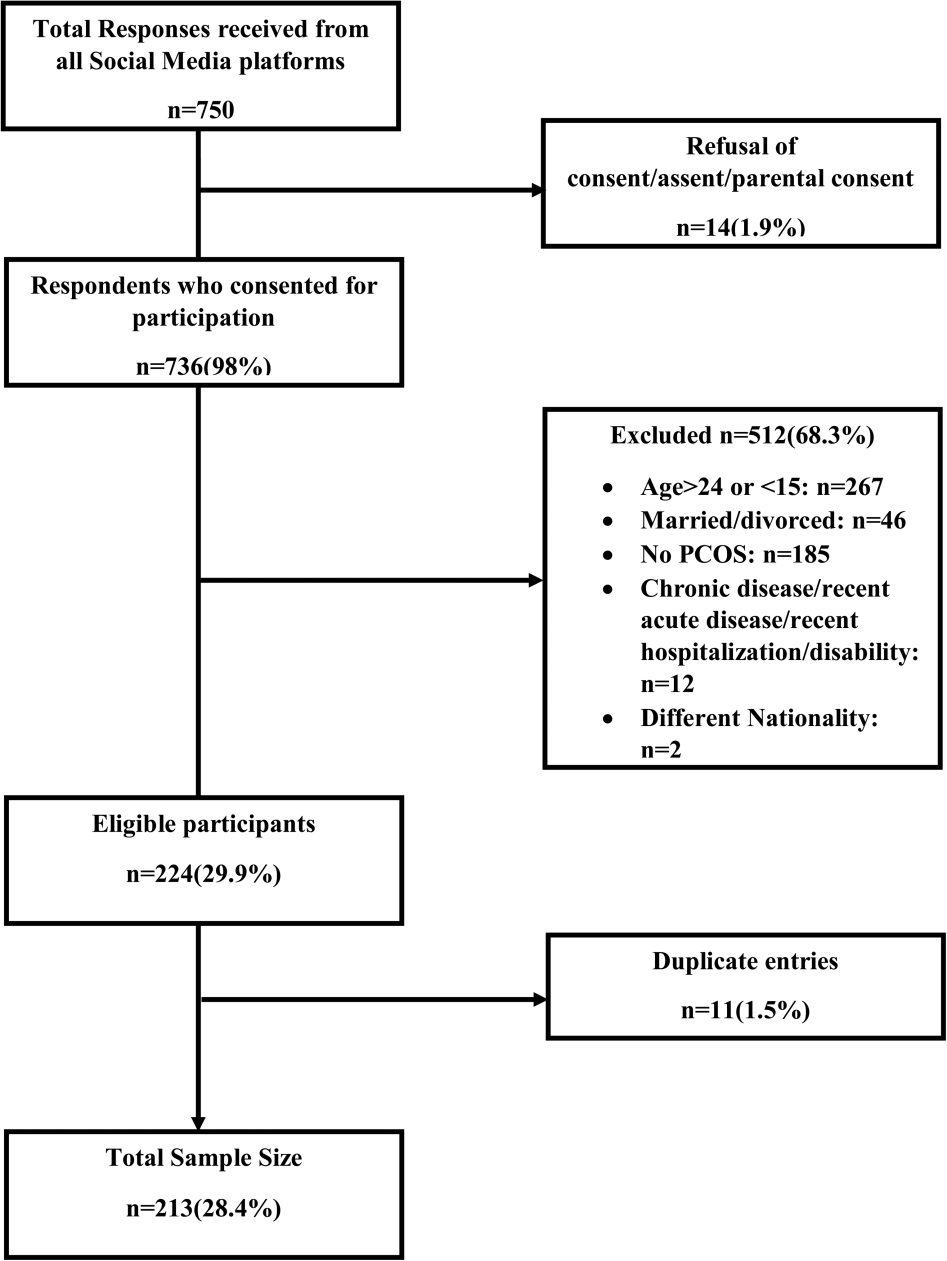
Flowchart of study participants.

The Google Form comprised of consent, screening form, and questionnaire. Informed consent for participants ≥18 years, informed assent, and parental consent for participants <18 years was taken. Parents/guardians' email addresses and contact details were taken to verify parental consent. To assess the eligibility, a screening form was administered prior to the main questionnaire. Single (neither widowed nor divorced) 15–24 years native Pakistani females with self-reported PCOS were recruited. We formulated questions based on the Rotterdam criteria for evaluating self-reported PCOS (24). Participant who had either oligomenorrhea (menstrual cycles >35 days or <21 days)/amenorrhea (less than two menstrual cycles in the last six months) or clinical signs (at least 2 of the symptoms, hirsutism, acne, alopecia, and obesity/weight gain)/biochemical signs (as it was not a possibility to check the biochemical test reports hence the participant was asked if she previously had any laboratory tests that confirmed higher androgen levels or the diagnosis of PCOS) or polycystic ovaries on ultrasound (ultrasound reports couldn't be checked hence participant was asked if she underwent an ultrasound that confirmed the presence of polycystic ovaries). If any of the two aforementioned criteria were met and the participant reported being diagnosed by a doctor/gynecologist/endocrinologist as having PCOS (a question was asked for a previous diagnosis by a doctor/gynecologist/endocrinologist), then only the participant was included in the study. If the participant was not diagnosed by a clinician or did not meet the Rotterdam criteria, the participant was excluded. Respondents with any chronic disease (diabetes, hepatitis), last month's history of acute disease/infection (COVID-19, typhoid, dengue), recent hospitalization, and any physical disability were excluded as their depression, and disease-specific QOL status might be affected**.** Flowchart of participants with exclusion is given in Figure 1. Only if the respondent was eligible was she navigated to the structured questionnaire for data collection; otherwise, there was an option to submit the form. Also, in all the questions after the eligibility were marked mandatory on Google Forms so the participant couldn't submit the form before answering all the questions, this was done to prevent any incomplete data. Both Urdu and English versions of the questionnaire were available. We pretested the questionnaire at 5% (*n* = 21) of the study's total sample size to ensure relevancy and adequacy.

### 2.1. Measures

#### 2.1.1. QoL (outcome) by PCOS-Q

QoL was assessed by the PCOS-Q tool. This tool was constructed in 1998 to determine the disease-specific QoL in women with PCOS. It comprises 26 items divided into five domains, representing the major manifestations of PCOS. These five domains include emotional health (8 items), menstrual abnormalities (4 items), hirsutism (5 items), infertility (4 items), and obesity (5 items). In infertility, domain items were modified to assess fears and concerns of infertility. Each question was answered by choosing a Likert scale response with seven options from 1 (always) to 7 (never). All responses were taken based on the past two weeks' experience or last menstrual period (25). Lower scores indicate poor QOL (25). In our study, Cronbach's alpha for the overall tool was 0.92, indicating high consistency and reliability. PCOSQ has been used internationally (6, 7) and regionally (10) in youth but not in Pakistan; hence its content validity was done by the experts.

#### 2.1.2. Depressive symptoms (primary exposure) by CES-D

Depressive symptoms were assessed by the CES-D tool. This tool is one of the most common self-reported tools used in youth to assess the current level of depressive symptomatology. Its main components include depressed mood, feelings of guilt and worthlessness, psychomotor retardation, feelings of helplessness and hopelessness, loss of appetite, and sleep disturbance. Each component of this scale has a few items, making it a total of 20 items. There are 4 responses for each question on the Likert scale ranging from never to all of the time. Participants were asked to answer the questions based on the past 1-week experience (14). Depression was taken as a categorical variable as a definite cutoff equal to or greater than 16 was considered indicative of depressive symptoms. This cutoff has been previously implemented in our settings (26). The CES-D tool's initial reliability in all ages showed high internal consistency with a Cronbach alpha of 0.85. Multiple preceding studies also depicted this scale's high internal consistency with Cronbach alpha greater than 0.8 (27). In our study, the CES-D tool was deemed highly consistent with the Cronbach alpha of 0.92. This tool has been used and validated in Pakistan on youth; therefore, it seemed most feasible to be used in this study (26).

#### 2.1.3. Other covariates

Sociodemographic characteristics including age, province, ethnicity, BMI, education, and the employment status of the parents were determined. Medical and family history consisting of PCOS duration, family history of PCOS, and infertility were also assessed. The frequency of engagement in physical exercises and any form of tobacco indulgence was determined as a part of health-related factors. Environmental characteristics including parental pressure/criticism due to PCOS symptoms conflicted parental relationship, type of other stressors (academic or work-related), societal pressure to improve the appearance that is affected due to PCOS, and level of satisfaction with the doctor/healthcare giver who is treating PCOS were evaluated.

### 2.2. Sample size

Power estimation of 80% and a level of significance of 0.05 was chosen for this study. We identified several studies to obtain the mean score and standard deviation of overall QOL in depressed and non-depressed PCOS women (11, 12). A minimum total of 125 women with self-reported PCOS were required to assess the association of depression and QOL to achieve 80% power and to detect a difference of 4.1 units in mean overall QOL scores between depressed and non-depressed PCOS youth at a significance level of 0.05 and at 1:4 ratio (non-depressed vs. depressed). As there was hardly any literature reporting the burden of depression in Pakistani youth with PCOS, we assumed a *post hoc* division of non-depressed vs. depressed at various ratios. A ratio of 1:4 (non-depressed vs. depressed) gave the maximum sample size; hence it was selected. After adding a 20% refusal rate due to a web-based approach, the minimum sample required for this study was 150 youth with PCOS. However, to obtain valid estimates and increase the power of the study, 213 participants were evaluated and analyzed in this study.

### 2.3. Statistical analysis

Analysis was done using STATA 16.0. Mean, and standard deviation was reported for normally distributed quantitative variables, whereas the median and interquartile range was reported for skewed variables. Frequency and percentages were reported for categorical variables. Simple and multiple linear regressions were used to assess predictors of QOL Crude and adjusted regression coefficients and 95% confidence intervals were reported. The presence of biologically plausible interaction was also evaluated at a significance level of <0.1.

### 2.4. Ethical considerations

A unique identifier number was assigned to each participant to ensure anonymity and confidentiality. Ethical approval was taken from The Aga Khan University Ethical Review Committee (ERC) AKUH ERC Ref #: 2020-3576-10343. All procedures performed in studies involving human participants were in accordance with the ethical standards of the institutional committee. Prior to administration of the questionnaire, electronic informed consent for participants ≥18 years was taken. In participants <18 years, electronic assent and parental/guardian consent was taken. Parental/guardian consent was verified by the contact details provided.

## 3. Results

### 3.1. Baseline characteristics of participants

The baseline characteristics of participants are given in Table 1.

**Table 1:** Baseline characteristics of Pakistani youth with PCOS stratified by age.

Baseline characteristics	Total *n* = 213 *n* (%)	15–19 years 36 (17) *n* (%)	>19–24 years 177 (83) *n* (%)	*p*-value
**SOCIODEMOGRAPHIC CHARACTERISTICS**				
Age*	21.4 (1.9)	18 (1.07)	22.04 (1.25)	<0.001
BMI**	24.7 (21.45, 29.3)	26.9 (22.7, 26.89)	24.1 (21.2, 29.3)	NS
**Province**				
Sindh	137 (64.3)	26 (72.2)	111 (62.7)	NS
Punjab	52 (24.4)	9 (25)	43 (24.3)	
Others	24 (11.3)	1 (2.8)	23 (13)	
**Ethnicity**				
Sindhi	47 (22.1)	9 (25)	38 (21.5)	NS
Punjabi	76 (35.7)	9 (25)	67 (37.8)	
Urdu speaking	40 (18.8)	11 (30.6)	29 (16.4)	
Others	50 (23.4)	7 (19.4)	43 (24.3)	
**Educational status**				
Matric/intermediate	66 (30.9)	29 (80.6)	37 (20.9)	<0.001
Graduate/post-graduate	147 (69.1)	7 (19.4)	140 (79.1)	
**Current employment status**				
Student	154 (72.3)	34 (94.4)	120 (68)	<0.001
Professional (employed or looking for job)	59 (27.7)	2 (5.6)	57 (32)	
**Socioeconomic status**				
Low	26 (12.2)	5 (14)	21 (12)	NS
Middle	155 (72.7)	25 (69)	130 (73)	
Status	32 (15.1)	6 (17)	26 (15)	
**Medical history, family history, and other health-related characteristics**				
Depressive symptoms	172 (80%)	29 (81)	143 (80.7)	NS
PCOS duration (years)**	2.5 (1, 4)	1.5 (0.6, 3)	3.3 (1, 5)	0.01
Family history of PCOS	59 (27.7)	14 (38.9)	45 (25.4)	NS
Family history of infertility	26 (12.2)	6 (16.7)	20 (11.3)	NS
No indulgence in physical exercise	52 (24.4)	6 (16.7)	46 (26)	NS
Tobacco usage in any form	18 (8.4)	5 (13.9)	13 (7.5)	NS
**Environmental characteristics**				
Academic/work stress	127 (59.6)	22 (61.1)	105 (59)	NS
Unjust criticism by parents in last 30 days related to PCOS	97 (45.5)	18 (50)	79 (44.6)	NS
Conflicted parental relationship	79 (37.1)	13 (36.1)	66 (37.3)	NS
Pressure from others to improve appearance	137 (64.3)	28 (77.8)	109 (61.6)	NS
Dissatisfaction from the doctor/healthcare giver treating PCOS and its symptoms	154 (72.3)	29 (80.6)	125 (70.6)	NS

BMI, body mass index; NS, non-significant; PCOS, polycystic ovarian syndrome.

* Mean (Standard Deviation).

** Median (IQR).

#### 3.1.1. Sociodemographic characteristics

A total of 213 Pakistani youth with PCOS were analyzed in this study. Almost 17% (36) of the participants were in 15–19 years age bracket whereas 83% (*n* = 177) were in >19–24 years age bracket. Only significant differences were seen in age, educational status and employment between both age brackets (*p* < 0.05). The mean age of the participants was 21.4 + 1.9 years. All participants' BMI was within the median (IQR) of 24.7 (21.45, 29.3). Most of the participants belonged to the province of Sindh (64.3%), followed by Punjab (24.4%). More than one-third of the respondents had Punjabi (35.6%), preceded by Sindhi (22.1%) ethnicities. The majority of the study participants had graduation/post-graduation as their maximum level of education (69.0%), at the time of the study were students (72.3%) and belonged to middle-class backgrounds (72.7%) (Table 1).

#### 3.1.2. Medical history, family history, and other health-related characteristics

There was no significant difference between medical history, family history and health related characteristics between participants of ages 15–19 years and >19–24 years (*p* > 0.05). Only PCOS duration differed between both age brackets (*p* < 0.05). All of the study respondents had PCOS duration since the first diagnosis within the median (IQR) of 2.5 years (1, 4). Almost 27.7% of the participants reported a family history of PCOS, whereas almost 12% reported having a family history of infertility. Approximately three-fourth (75.5%) of the participants reported regular indulgence in some physical activity. Moreover, almost 8% of the respondents reported occasional usage of any tobacco variant (Table 1).

#### 3.1.3. Environmental characteristics

There was no difference in environmental characteristics were seen between age brackets of 15–19 years and >19–24 years (*p* > 0.05). The majority of the study participants reported going through either academic or work-related stress (60.0%), facing immense societal pressure to improve their appearance affected due to PCOS (64.3%) and dissatisfaction and frustration from the doctor or healthcare giver treating PCOS and its symptoms (72.0%). Less than half (45.5%) of the respondents reported facing unjust criticism from their parents related to PCOS and its manifestations in the last 30 days. Furthermore, approximately 37% of the respondents perceived their relationship with their parents as too conflicted, adding on to more stress (Table 1).

### 3.2. Comparison of quality of life in participants as per depression status and age

More than three-fourths (80%) of the participants screened positive for depressive symptoms.

Mean QOL and respective domain scores are given in Table 2. The overall mean QOL score was 2.9 + 1.1. A statistically significant difference was seen between mean overall QOL and individual domain scores of depressed and non-depressed participants. In depressed participants, the most distressing domain was obesity/weight gain with a mean score of 2.5 + 1.6, followed by emotional health 2.8 ± 1.1. The least distressing domain in depressed participants was hirsutism, with a mean score of 3.1 ± 1.9. A similar trend was seen in non-depressed participants, with obesity/weight gain (2.7 ± 1.5) and emotional health domain (3.4 ± 1.4) being the most distressing domains. However, the third most distressing domain in depressed participants was menstrual abnormalities (2.8 ± 1.3), whereas, in non-depressed participants, it was the infertility domain (3.4 ± 1.7). The mean overall QOL score was lower in depressed (2.8 ± 1.0) than non-depressed participant (3.4 ± 1.3). Depressed participants scored significantly lower in all domains of QOL and overall QOL score than non-depressed participants indicating the lower quality of life in depressed participants (*p*-value <0.001). Statistical significance of the difference in mean scores was determined using two independent sample *t*-test (Table 2). In participants between ages 15–19 years, obesity (2.1 ± 1.3) followed by emotional health (2.8 ± 1.2) are the most distressing domains whereas infertility (3.4 ± 1.8) is the least distressing domain. In ages >19–24 years, the least distressing domain was hirsutism (3.2 ± 2). There was no significant difference in overall QOL and each domain of QOL between age brackets of 15–19 years and >19–24 years (*p* > 0.05) (Table 2).

**Table 2:** Mean overall QOL and domains score as per the depressive symptoms of Pakistani youth with PCOS stratified by ages (15–19 years) and (>19–24 years).

Quality of life domains	Total *n* = 213 Mean (SD)	15–19 years 36 (17%) Mean (SD)	>19–24 years 177 (83%) Mean (SD)	*p*-value	Depressive symptoms 172 (80%) Mean (SD)	No depressive symptoms 41 (20%) Mean (SD)	*p*-value
Obesity	2.5 (1.6)	2.1 (1.3)	2.6 (1.7)	0.13	2.5 (1.6)	2.7 (1.5)	<0.001
Emotional health	2.9 (1.2)	2.8 (1.2)	2.9 (1.3)	0.89	2.8 (1.1)	3.4 (1.4)	<0.001
Menstrual abnormalities	3.1 (1.5)	3.3 (1.7)	3 (1.4)	0.21	2.8 (1.3)	3.7 (1.7)	<0.001
Infertility	3.1 (1.6)	3.4 (1.8)	3.1 (1.6)	0.24	3.0 (1.6)	3.4 (1.7)	<0.001
Hirsutism	3.1 (1.9)	2.96 (1.9)	3.2 (2.0)	0.5	3.1 (1.9)	3.8 (2.1)	<0.001
Overall Quality of life	2.9 (1.1)	2.9 (1.1)	2.9 (1.1)	0.97	2.8 (1.0)	3.4 (1.3)	<0.001

Lower the score, the poorer the quality of life.

Significance (*p*-value) assessed by two-independent samples *t*-test.

QOL, quality of life; SD, standard deviation.

### 3.3. Quality of life and its predictors

Predictors of QOL are given in Table 3. The findings suggest that the mean QOL scores were lower in respondents who faced immense societal pressure to improve appearance affected by PCOS compared to participants who met no such pressure was −9.3 (−16.8, −1.9). Parental criticism due to PCOS was another factor negatively influencing the mean QOL score −7.9 (−14.8, −1.1). Also, the mean QOL score was lower among professionals (either employed or awaiting job) than current students (adjusted β:−91.8 CI: −16.6, −1.7). The difference in mean QOL score between participants with graduation/post-graduation and participants with matriculation/intermediate as their highest education status was −7.6 (−15, −0.3). BMI was also found to be negatively associated with the mean QOL score as with every kg/m^2^ increase in BMI; the estimated mean QOL score was decreasing by 0.5 (−0.9, −0.1). Furthermore, the difference in mean QOL scores between respondents who belonged to middle-class backgrounds and participants with high socioeconomic status was −14.16 (−23, −5.3) (Table 3).

**Table 3:** Crude and adjusted regression coefficients along with 95% confidence intervals for predictors influencing QOL in Pakistani youth with PCOS.

Characteristics	Crude β (95% CI)	Adjusted β (95% CI)
Depressive symptoms	−12.8 (−22.2, −3.3)	–
Societal Pressure affected by PCOS to improve appearance	−20.1 (−27.6, −12.7)	−9.3 (−16.8, −1.9)
Dissatisfaction with doctor/healthcare giver treating PCOS and its symptoms	−20.7 (−28.7, −12.7)	–
Parental criticism related to PCOS	−15.5 (−22.8, −8.2)	−7.9 (−14.8, −1.1)
Working status		
Professionals (currently employed)	−16.9 (−25.1, −8.8)	−9.1 (−16.6, −1.7)
Education		
Graduate/postgraduate	−12.2 (−20.3, −4.2)	−7.6 (−15, −0.3)
BMI	−0.6 (−1.1, −0.2)	−0.5 (−0.9, −0.1)
Socioeconomic status		
Low	−12.1 (−26.9, 2.2)	−11.4 (−23.9, 0.9)
Middle	−14.1 (−24.6, −3.4)	−14.1 (−23, −5.3)
Duration of PCOS (in years) since diagnosis	−2.1 (−3.5, −0.8)	–
Family History of PCOS	−4.9 (−13.3, 3.5)	–
• Depressive symptoms with a year increase in the duration of PCOS since the first diagnosis		−25.1 (−36.6, −13.6)
• No depressive symptoms with a year increase in the duration of PCOS since the first diagnosis		−5.3 (−9.1, −1.5)
• No Family history of PCOS and satisfaction with treatment giver		–
• Family history of PCOS and satisfaction with treatment giver		7.9 (−5.5, 21.3)
• No Family history of PCOS and dissatisfaction with treatment giver		−5.9 (−14.6, 2.8)
• Family history of PCOS and dissatisfaction with treatment giver		−17.4 (−26.1, −8.8)

Adjusted *R*^2^ for final model: 0.37.

The *p*-value for the final model <0.001.

F statistic for final model: 8.96.

**Figure 2:**
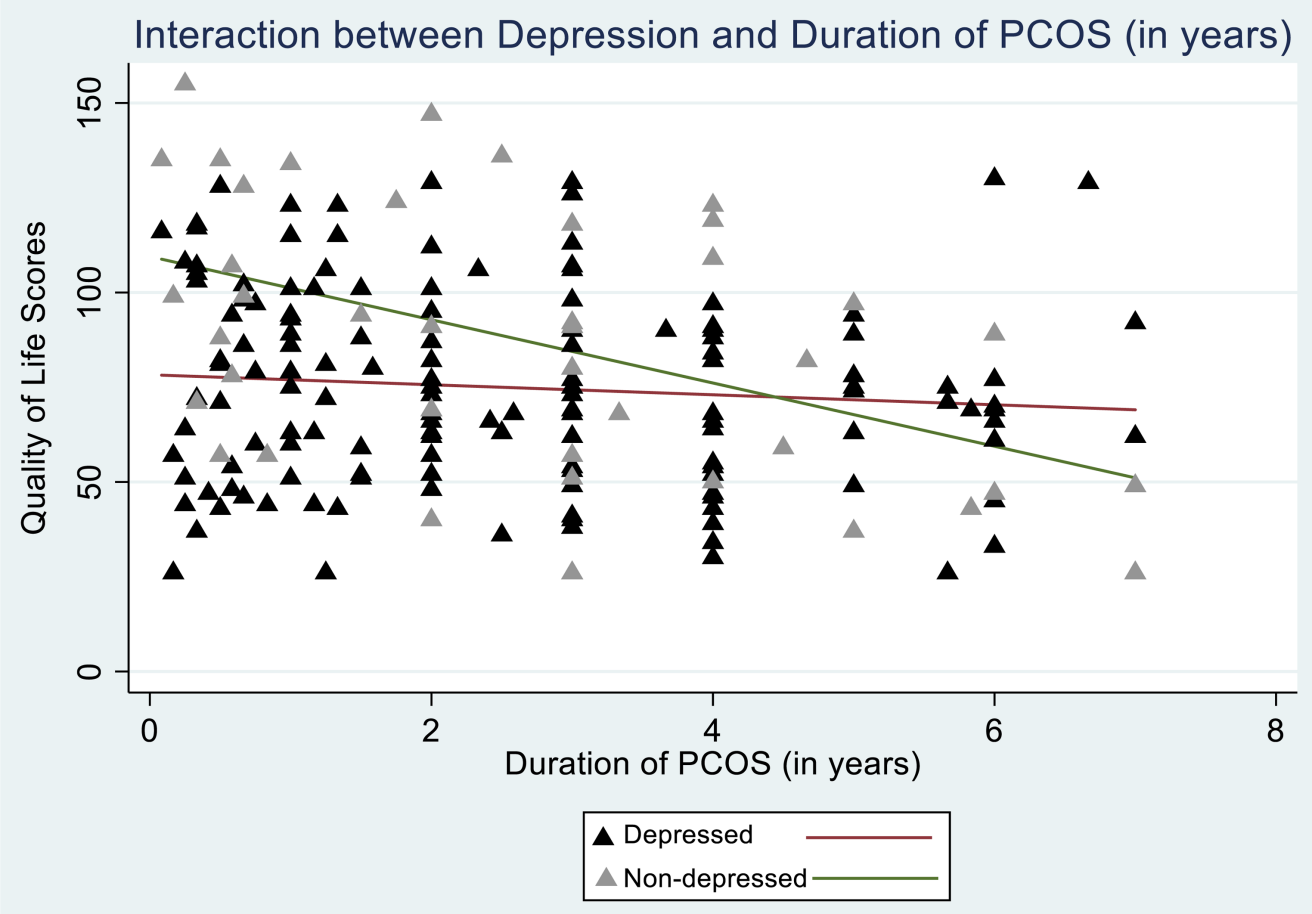
Interaction between depressive symptoms and duration of PCOS since the first diagnosis (in years).

Interaction between depression status and PCOS duration since the first diagnosis (Figure 1) was found to be significant in the final model, indicating that the estimated mean overall QOL score decreases by 25.1 (−36.6, −13.6) for every year increase in PCOS duration since the first diagnosis among participants screened positive for depression. Among non-depressed participants, the estimated mean overall QOL scores decrease by 5.3 (−9.1, −1.5) with every year increase in PCOS duration since the first diagnosis (Table 3). Interaction graph is given in Figure 2.

**Figure 3:**
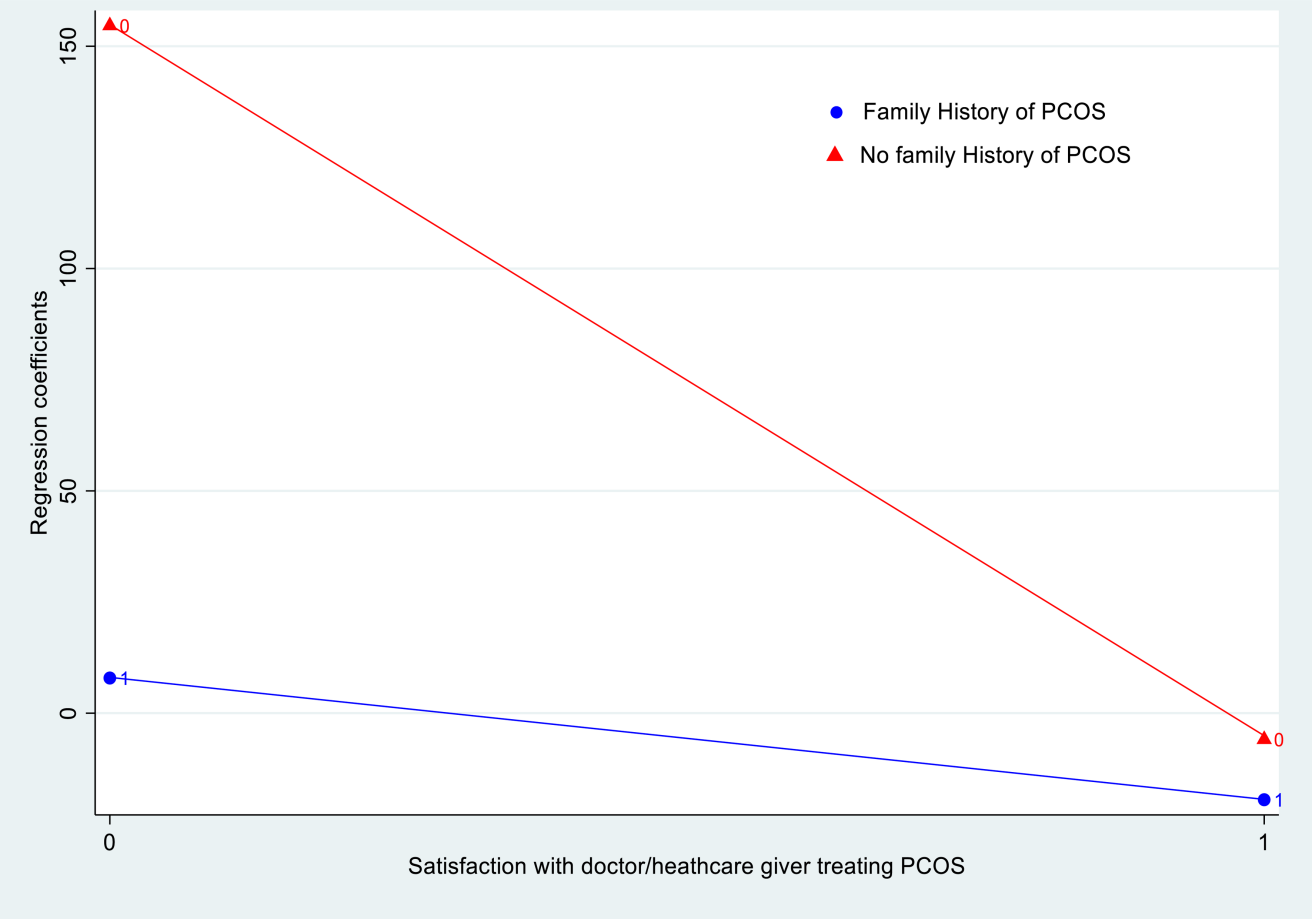
Interaction between family history of PCOS and dissatisfaction with healthcare provider treating PCOS and its symptoms.

Another significant interaction was seen between family history of PCOS and dissatisfaction with the doctor/healthcare giver treating PCOS symptoms (Figure 3). Among participants who had a family history of PCOS and were dissatisfied with their doctor/healthcare giver treating PCOS and its symptoms, the estimated mean overall QOL decreased by 17.4 (−26.1, −8.8) compared to youth with no family history of PCOS and were satisfied with their doctor/healthcare giver in treating PCOS and its symptoms (Table 3). Interaction graph is given in Figure 3.

Overall, 37% of the variability in the mean QOL score of the participants was explained by the predictors mentioned above and interaction.

## 4. Discussion

The present study explored the association between depressive symptoms and QOL and factors associated with QOL in Pakistani youth with PCOS. QoL was significantly associated with social pressure to improve appearance affected by PCOS, constant parental criticism due to PCOS, education status, working status, socioeconomic status, the interaction between depressive symptoms and duration of PCOS, and interaction between family history of PCOS and satisfaction with the healthcare giver treating PCOS and its symptoms.

Overall QOL of the respondents was on the lower side compared to literature depicting that QOL is indeed affected due to PCOS (28). The domain of obesity/weight gain was found to be the most distressing domain of QOL Studies from Canada, England, and Brazil, showed alignment with this finding and reported domain of obesity having the most significant impact on QOL (6, 17, 29). Probable reasons for this finding might be that as youth are conscious about physical appearance, any increase in weight leads to a negative body image, resulting in decreased body satisfaction and impaired QOL Moreover, the societal idealization of thin bodies in our settings further adds to distress, ultimately reducing QOL (30). However, regionally in Iran, hirsutism, menstrual abnormalities, and infertility were reported as the most distressing factors influencing QOL in women with PCOS (31, 32). Differences in the socio-cultural context can explain this contrast.

One of the most significant findings of this study was a reduction in overall QOL as well as individual domain scores in depressed respondents compared to their non-depressed counterparts. Our study showed agreement with the study by Greenwood et al. (12). A possible explanation for inferior QOL in depressed participants might be because depression causes individuals to perceive their symptoms with more gravity leading to self-victimization and ultimately reduced QOL (12). Moreover, in a chronic disease like PCOS, where lifestyle modification is a prerequisite for producing a change, depression might lead to loss of motivation, increasing the severity of the symptoms and eventually worsening QOL.

Social pressure to improve appearance affected by PCOS was a significant contributor to QOL in our study. A similar study in Iran replicated this finding and reported that females who faced constant criticism by others to improve appearance had poor QOL (33). A possible explanation of this finding could be that continuous social pressure results in decreased coping and self-worth, leading to disturbed QOL (33).

Parental criticism due to PCOS was another predictor of QOL. Respondents who faced constant criticism and lack of empathy and support from their parents relative to PCOS and its symptoms reported reduced QOL (34). Parent's constant judgments leading to the child's introverted character and self-isolation might explain the reduction of QOL (34).

The educational status of the participants was also associated with QOL. Literature indicates that an individual becomes more aware of the environment and interpersonal differences with increasing educational level, leading to increased self-criticism and self-dissatisfaction, eventually disturbing QOL (34, 35). However, conflicting results are reported by another study, which reported that the lower the educational level, the worse is the QOL (31).

BMI was negatively associated with overall QOL. This was evidenced by another study which suggested negative correlation of BMI with QOL (36). Conventional norms suggesting being skinny or thin as definition of beautiful might explain why gain in weight negatively affects QOL.

Participants in our study who were employed reported poor QOL. Similar studies replicated this finding and reported lower QOL in employed women than homemakers/students (16, 31). A possible justification for this finding might be that interpersonal interaction is increased in participants who are employed. They face more judgment and scrutiny from their colleagues leading to an inferiority complex and ultimately poor QOL (16).

Socioeconomic status was another determinant of QOL in our settings. Participants belonging to low and middle-class backgrounds had poor QOL. This finding was comparable with another study, which suggested that individuals with high socioeconomic standing had better QOL (16). For this association, probable justification might be that individuals with high financial standing can afford to seek the best and most expensive treatment available and implement relevant lifestyle modifications, leading to controlled symptoms and better QOL (16). However, the literature is inconsistent with this finding (12).

One of the most key findings of this study was the interaction between depressive symptoms and PCOS duration. It has been well-established in the literature that QOL gets poorer with increasing PCOS duration since the first diagnosis (37). Also, the association between depression and inferior QOL has been evidenced in previous studies (12, 36, 38–40). Our study showed that depressed participants had poor QOL with increasing duration of PCOS. Long term implications and esthetic changes due to PCOS are already very distressing; depressive tendencies might further intensify this distress and lead to self-victimization, loss of motivation, and hopelessness, eventually affecting QOL.

Interaction between family history of PCOS and satisfaction with the healthcare giver treating PCOS and its symptoms was another important finding of this study. Literature evidences satisfaction with physicians treating PCOS as a significant determinant of QOL (17). Also, the family history of PCOS is known to play a part in influencing QOL (10). Individuals with a family history of PCOS are already in distress because they have seen their family member going through its symptoms and long-term consequences. Furthermore, if they perceive that their healthcare giver is unable to understand and resolve their symptoms and is not considering the psychological impact of PCOS, in that case, it can add on to further distress and worsening of QOL (17).

To the best of our knowledge, this is the first study to explore QOL and its factors in Pakistani youth with PCOS. Content validation of tools to ensure cultural and contextual relevancy was also one of the study's major strengths. One of the few limitations of this study includes under or over-reporting of the status of PCOS by the respondents, which couldn’t be rectified due to the unavailability of Ultrasound and laboratory reports. We used Rotterdam criteria which included ultrasound for diagnosis of PCOS which is not preferred in adolescents ≤19 years of age, hence it is also a limitation. Another limitation was the subjectivity of exposure (Depression) and outcome (QOL). BMI was calculated by the weight and height given by the participants, hence there is subjectivity in the variable of BMI and should be interpreted with caution. The tool used for assessing QOL was PCOS specific and didn't cater to overall QOL which might be another limitation. We did not take into consideration medications used by participants for PCOS or its symptoms and other parameters including prolactin, which might have added to the study. Lastly, the generalizability of the study is limited to educated youth with diagnosed PCOS, internet access and from good socioeconomic status with access to internet device. Half of the women with PCOS might not have been included because of limited access to the internet'.

## 5. Conclusion

Our study's findings indicate that depressive participants with an increasing duration of PCOS are associated with poor QoL Counselling and cognitive-behavioural therapies to treat psychological morbidities may help improve the overall QOL of PCOS women. Moreover, counselling and training health professionals and families to understand the implications and mental stresses the PCOS women face might also better QOL Societal awareness programs to impart knowledge about PCOS and its clinical stigmata may also help reduce constant judgments and scrutiny faced by PCOS women. The findings of this study must be interpreted carefully as it is reflective of youth with internet access and diagnosed PCOS by a healthcare professional.

## Data Availability

The original contributions presented in the study are included in the article, further inquiries can be directed to the corresponding author.
